# Effects of *Helicobacter suis* γ- Glutamyl Transpeptidase on Lymphocytes: Modulation by Glutamine and Glutathione Supplementation and Outer Membrane Vesicles as a Putative Delivery Route of the Enzyme

**DOI:** 10.1371/journal.pone.0077966

**Published:** 2013-10-16

**Authors:** Guangzhi Zhang, Richard Ducatelle, Frank Pasmans, Katharina D’Herde, Liping Huang, Annemieke Smet, Freddy Haesebrouck, Bram Flahou

**Affiliations:** 1 Department of Pathology, Bacteriology and Avian Diseases, Faculty of Veterinary Medicine, Ghent University, Merelbeke, Belgium; 2 Department of Basic Medical Sciences, Faculty of Medicine and Health Sciences, Ghent University, Ghent, Belgium; 3 Division of Swine Infectious Diseases, State Key Laboratory of Veterinary Biotechnology, Harbin Veterinary Research Institute, Chinese Academy of Agricultural Sciences, Harbin, China; East Carolina University School of Medicine, United States of America

## Abstract

*Helicobacter* (H.) *suis* colonizes the stomach of the majority of pigs as well as a minority of humans worldwide. Infection causes chronic inflammation in the stomach of the host, however without an effective clearance of the bacteria. Currently, no information is available about possible mechanisms *H. suis* utilizes to interfere with the host immune response. This study describes the effect on various lymphocytes of the γ-glutamyl transpeptidase (GGT) from *H. suis*. Compared to whole cell lysate from wild-type *H. suis*, lysate from a *H. suis ggt* mutant strain showed a decrease of the capacity to inhibit Jurkat T cell proliferation. Incubation of Jurkat T cells with recombinantly expressed *H. suis* GGT resulted in an impaired proliferation, and cell death was shown to be involved. A similar but more pronounced inhibitory effect was also seen on primary murine CD4^+^ T cells, CD8^+^ T cells, and CD19^+^ B cells. Supplementation with known GGT substrates was able to modulate the observed effects. Glutamine restored normal proliferation of the cells, whereas supplementation with reduced glutathione strengthened the *H. suis* GGT-mediated inhibition of proliferation. *H. suis* GGT treatment abolished secretion of IL-4 and IL-17 by CD4^+^ T cells, without affecting secretion of IFN-γ. Finally, *H. suis* outer membrane vesicles (OMV) were identified as a possible delivery route of *H. suis* GGT to lymphocytes residing in the deeper mucosal layers. Thus far, this study is the first to report that the effects on lymphocytes of this enzyme, not only important for *H. suis* metabolism but also for that of other *Helicobacter* species, depend on the degradation of two specific substrates: glutamine and reduced glutatione. This will provide new insights into the pathogenic mechanisms of *H. suis* infection in particular and infection with gastric helicobacters in general.

## Introduction

*Helicobacter pylori* can cause gastritis, peptic ulcer disease, gastric adenocarcinoma and mucosa-associated lymphoid tissue (MALT) lymphoma in humans [1,2]. It is, however, not the only bacterial pathogen capable of colonizing the human gastric mucosa. Indeed, gastric non*-H. pylori* helicobacters (NHPH) have also been detected in humans and these bacteria are capable of causing disease in both humans and animals [3-11]. *H. suis* has been shown to be the most prevalent gastric NHPH in humans [3]. Similar to *H. pylori*, *H. suis* generally causes a life-long infection, suggesting that the bacterium possesses immune suppressing properties.

Lymphocyte responses are involved in a wide range of immunoregulatory activities, both *in vivo* and *in vitro* [12]. So far, no information is available on the influence of *H. suis* virulence determinants on the function of lymphocytes. For *H. pylori*, several factors have been described having an effect on the host lymphocyte response, including the vacuolating cytotoxin (VacA) and *H. pylori* GGT [13-15]. The former is absent in *H. suis* [16]. The latter enzyme, synthesized as a precursor enzyme with a molecular weight (MW) of ~60 kDa, followed by processing into a large (~40 kDa ) and small (~20 kDa ) subunit [17], is also produced by a number of other *Helicobacter* species, including *H. suis*, and the enzyme has been shown to play an important role during the metabolism of extracellular L-glutamine (L-Gln) and reduced glutathione (GSH) [17,18]. Degradation of GSH, an important antioxidant, by GGT results in the development of extracellular oxygen radicals, leading to oxidative damage of epithelial cells, or inhibition of cellular proliferation [17,19,20]. In contrast, Gln, another substrate of GGT, is a major metabolic fuel for rapidly dividing cells, including enterocytes and immunologically challenged lymphocytes [21,22]. In addition, regulation of L-Gln utilization seems to be an important component of T cell activation and the development of an immune response and Gln is also a key regulator of gene expression and cell signalling pathways [23,24]. Currently, no information exists regarding a possible regulatory effect of L-Gln or GSH (supplementation) on the proliferation of lymphocytes affected by the GGT of gastric helicobacters.

It has been demonstrated that the GGT secreted from gastric helicobacters as well as other secreted factors such as the VacA from *H. pylori* can access the lymphocytes in the lamina propria. These secreted factors may affect the lymphocyte function in a direct and indirect manner, for instance by inflicting damage to epithelial cells, resulting in small epithelial defects [3,17,25,26]. Interestingly, *H. pylori* outer membrane vesicles (OMV) have been shown to contain the *H. pylori* GGT [27] and they have been shown to be internalized by epithelial cells [28]. In general, OMV are released by Gram-negative bacteria under natural conditions *in vitro* or in infected tissue *in vivo*, and they can act as a delivery vehicle of virulence factors to reach a distant target [29-32]. Thus far, no information is available on the formation of *H. suis* OMV, the content thereof, their internalization by epithelial cells and the putative delivery of bacterial components, such as the *H. suis* GGT, to the deeper mucosal layers. 

In the present study, Jurkat T cells as well as murine splenocyte subsets (CD4^+^ T cell, CD8^+^ T cell, CD19^+^ B cell) were used as cell models to investigate the immunosuppressive effect of *H. suis* GGT through the action on its substrates. AGS cells, intestinal porcine epithelial (IPEC-J2) cells, and human Caco-2 cells were used to investigate the putative translocation of GGT, present in *H. suis* OMV, across an epithelial cell monolayer. 

## Materials and Methods

### Animals

For isolation of splenic lymphocytes, female specific-pathogen-free (SPF) 4-6-week-old BALB/c mice were purchased from Harlan NL (Horst, The Netherlands). Housing and euthanasia of experimental animals were approved by the Ethical Committee of the Faculty of Veterinary Medicine, Ghent University, Belgium (EC2012/156). 

### Construction of a *H. suis ggt* isogenic mutant strain

Deletion of *H. suis ggt* was introduced by allelic exchange using pBluescript II SK (+) phagemid vector (Agilent Technologies, California, USA) in which ~650 bp of the 5′ –end and ~750 bp of the 3′ –end of the target gene and the chloramphenicol resistance gene from pUOA14 [18,33] were ligated through a PCR-mediated strategy [34,35]. All primers used for PCR-mediated construction of the recombinant plasmid are shown in table 1. The resultant plasmid was amplified in XL1-Blue MRF′ *E. coli* (Agilent Technologies) and used as a suicide plasmid in *H. suis* strain HS5, isolated from the stomach of a sow. Transformation of *H. suis* strain HS5 was perfomed by electroporation as described for *H. felis* [36] with some modifications. Briefly, 1.5 µg suicide plasmid was used for electroporation. Then, the *H. suis 5 ggt* mutant strain (HS5Δ*ggt*) was first cultured for 2 days on biphasic *Brucella* culture plates without chloramphenicol, as described previously [37]. Subsequently, bacteria were transferred onto biphasic *Brucella* culture plates supplemented with chloramphenicol (20 µg/mL) for 4 days, after which they were finally selected on biphasic *Brucella* plates supplemented with chloramphenicol (30 µg/mL) for 7-14 days. The site of recombination was verified by a GGT activity assay [17], PCR and nucleotide sequencing. 

**Table 1:** Primers used for construction of a *H. suis ggt* isogenic mutant strain (HS5Δ*ggt*).

**Primer name**	**Sequence (5′ - 3′)**	**Primer use**
pBlue linear Fwd 1	GGGGATCCACTAGTTCTAGAGCG	Linearization of plasmid
pBlue linear Rev1	CGGGCTGCAGGAATTCGATATCAAG	Linearization of plasmid
HsGGT_flank_fusion1F	CTTGATATCGAATTCCTGCAGCCCGGAGGCGTTGCACAATAGCTTTAGGG	Amplification *H. suis ggt* and partial up- and downstream flanking genes
HsGGT_flank_fusion1R	GCCGCTCTAGAACTAGTGGATCCCCATAAAACCAGTTAGGCTGGGCAAAG	Amplification *H. suis ggt* and partial up- and downstream flanking genes
pBluelinear_Hsggtflank1F	CCACGCAAGGAATTTTAAATGCAAC	Linearization of the recombinant plasmid
pBluelinear_Hsggtflank1R	GATCTCCTCAAATTTTAAAAAATACGC	Linearization of the recombinant plasmid
Hschloram_fusion_1F	GCGTATTTTTTAAAATTTGAGGAGATCTATCAACAAATCGGAATTTACGG	Amplification chloramphenicol resistance gene
Hschloram_fusion_1R	GCATTTAAAATTCCTTGCGTGGTTATTTATTCAGCAAGTCTTGTAA	Amplification chloramphenicol resistance gene
T7 prom3	TAATACGACTCACTATAGGG	Sequencing
M13R	CAGGAAACAGCTATGAC	Sequencing

### Recombinant expression and purification of *H. suis* γ-glutamyl transpeptidase

The expression and subsequent purification of recombinant *Helicobacter suis* γ-glutamyl transpeptidase (GGT) were performed as described previously [17]. Briefly, the enzyme was expressed in *E. coli* strain BL21-AI^TM^. Subsequently, the protein was purified to homogeneity by immobilized metal affinity chromatography (IMAC) on a Ni-sepharose column (His GraviTrap; GE Healthcare Bio-Sciences AB, Uppsala, Sweden) and gel filtration using a Superdex^TM^ 75 gel filtration column (GE Healthcare Bio-Sciences AB). The purified protein was stored at -80°C until further use.

### Preparation of *H. suis* outer membrane vesicles (OMV)

72-hour-old cultures of *H. suis* were harvested, and the bacteria were removed by centrifugation (12000 × g, 15 minutes, 4 °C). The supernatant fluid was subjected to ultracentrifugation (200000 × g, 2 hours, 4 °C) to recover the OMV. After two washing steps in Hank’s Balanced Salt Solution (HBSS), the OMV were stored at -70 °C until further use. The obtained OMV were visualized by a negative staining technique. Hereby a copper grid with formvar membrane was placed on top of a drop of OMV suspension for 10 seconds and counterstained with uranylacetate for 1 minute. After rinsing and drying the grids were analysed by Transmission Electron Microscopy (TEM). The presence of GGT activity in *H. suis* OMV was validated with a GGT activity assay as described previously [17].

### Cell cultures

Jurkat E6.1 cells (Human leukaemic T cell line; ECACC; Salisbury, UK) were cultured in RPMI 1640 with 5% (v/v) heat-inactivated fetal bovine serum (FBS; HyClone, Logan, UT, USA), 2 millimolar (mM) L-Gln (Invitrogen, Carlsbad, CA, USA) and penicillin (50 units/mL) and streptomycin (50 µg/mL) (Invitrogen) at 37°C with 5% CO_2_.

CD4^+^ and CD8^+^ T cells, as well as CD19^+^ B lymphocytes were isolated from mouse spleens using EasySep™ Mouse CD4^+^ and CD8^+^ T cell, and CD19^+^ B cell Enrichment Kits (StemCell Technologies, Grenoble, France). Culture was performed in RPMI 1640 containing 10% (v/v) FBS, 1 mM L-Gln, 50 micromolar (µM) 2-mercaptoethanol (Sigma-Aldrich St. Louis, MO, USA), penicillin (50 units/mL) and streptomycin (50 µg/mL) at 37°C with 5% CO_2_.

The culture conditions of AGS cells (a human gastric adenocarcinoma cell line), IPEC-J2 cells, and Caco-2 cells have been described elsewhere [17,38,39]. Briefly, AGS cells were cultured in Ham’s F12 (Invitrogen; 1 mM glutamine) supplemented with 10% (v/v) FBS, penicillin (50 units/mL) and streptomycin (50 µg/mL). IPEC-J2 cells were cultured in Dulbecco’s Modified Eagle’s medium (DMEM; Gibco, Life Technologies, Paisley, Scotland) supplemented with 47% (v/v) Ham’s F12 medium (Gibco), 5% (v/v) FBS, 1% (v/v) insulin-transferrin-selenium-A supplement (ITS, Gibco), penicillin (50 units/mL), and streptomycin (50 µg/mL). Caco-2 cells were cultured in DMEM (Gibco) supplemented with 10% (v/v) FBS, 1 mM glutamine, 1% (v/v) non-essential amino acids (Gibco), penicillin (50 units/mL) and streptomycin (50 µg/mL). 

### Internalization of *H. suis* OMV by AGS, IPEC-J2 and Caco-2 cells

AGS, IPEC-J2 and Caco-2 cells were used to examine the putative internalization of *H. suis* OMV. AGS cells labeled with green CellTracker^TM^ (Invitrogen) were incubated for 4 hours with *H. suis* OMV labeled with red fluorescent Vybrant^®^ DiD (Invitrogen). AGS cells were fixed with 4% paraformaldehyde for 15 minutes, washed 5 times extensively with HBSS and analysed by confocal laser scanning microscopy for uptake of *H. suis* OMV. IPEC-J2 and Caco-2 cells were labeled with red fluorescent CellTracker Red CMTPX (Invitrogen) and incubated for 8 hours with *H. suis* OMV labeled with green fluorescent Vybrant^®^ DiO (Invitrogen). Subsequently, cells were fixed with 4% paraformaldehyde for 15 minutes, washed 5 times extensively with HBSS and analysed by confocal laser scanning microscopy for uptake of *H. suis* OMV.

### Translocation across a differentiated IPEC-J2 monolayer of active GGT present in *H. suis* OMV

In order to examine the putative translocation ability of active *H. suis* GGT contained in *H. suis* OMV across an epithelial cell monolayer, a translocation assay was performed as described elsewhere [40]. IPEC-J2 cells (1 × 10^4^ cells/250 µl/insert) were seeded on the apical side of the Transwell® polycarbonate membrane inserts with a pore size of 3.0 μm and a membrane diameter of 6.5 mm (Corning Costar Corp., Cambridge, MA, USA), and the basolateral side was filled with 1 mL fresh culture medium. Cell medium was refreshed every 2 to 3 days and cells were cultured for 3 to 4 weeks in order to allow differentiation to a complete monolayer as described elsewhere [39,40]. When differentiated, 100 µg (based on the total protein content) *H. suis* OMV were added to the apical compartment. After incubation for up to 48 hours (37°C; 5% CO_2_), the presence of GGT activity in the basolateral compartment was determined with a GGT activity assay [17]. The transepithelial electrical resistance (TEER) was measured before and after the incubation with *H. suis* OMV to assess the barrier integrity of the differentiatedepithelial cell monolayer as described previously [40].

### Cell proliferation assays

Jurkat T cells (4 × 10^4^/well), CD4^+^ and CD8^+^ T, and CD19^+^ B lymphocytes (1.5 × 10^5^/well) were cultured in 24-well or 96-well flat-bottom cell-culture plates (Greiner Bio One, Frickenhausen, Germany) as described above.

CD4^+^ and CD8^+^ T cells were stimulated by incubating the cells in wells of a microtiter plate that had been precoated with an anti-CD3 antibody (4 µg/mL and 8 µg/mL respectively, clone 145-2C11; eBioscience, Vienna, Austria) and in the presence of a soluble anti-CD28 antibody (2 µg/mL, clone 37-51; eBioscience). CD19^+^ B cells were stimulated by F(ab’)2 Goat anti-mouse IgM (12 µg/mL, Jackson Immunoresearch, West Grove PA, USA) and recombinant mouse IL-2 (100 U/mL, eBioscience).

All cells were incubated in the presence or absence of whole-cell lysate from wild-type *H. suis* strain HS5 and mutant *H. suis* strain HS5Δggt, as well as different concentrations of recombinant *H. suis* GGT for 24 - 72 hours, depending on the experiment and cell type. Cellular proliferation was determined by incorporation of [^3^H]-thymidine (Amersham ICN, Bucks, UK). In brief, all cells were pulse-labeled with 1 µCi [^3^H]-thymidine during the final 18 hours of experimental incubation, and then harvested onto glass fiber filters (Perkin-Elmer, Life Science, Brussels, Belgium). The incorporated radioactivity was detected using a β-scintillation counter (Perkin-Elmer).

### Evaluation of cell death (apoptosis and necrosis) by flow cytometry

Jurkat T cells (4 × 10^4^/well) were treated with 2 µg/mL recombinant *H. suis* GGT for 24 - 72 hours. Controls consisted of HBSS-treated Jurkat T cells. All samples were subjected to flow cytometric analysis (FCM) on a BD FACSCanto II flow cytometer with FACSDiva software (Becton Dickinson, Erembodegem, Belgium). 

 Propidium iodide (PI) staining was used to detect loss of plasma membrane integrity as a marker for necrosis. Briefly, cells were washed with HBSS, incubated with 1 µg/mL PI in HBSS for 15 minutes on ice, followed by FCM analysis. Staining for activated caspase-3 was performed to detect apoptosis. Briefly, cells were washed with HBSS, fixed with 4% paraformaldehyde for 10 minutes, and permeabilized with 0.1% Triton X-100 in HBSS for 2 minutes. Subsequently, cells were incubated with a primary rabbit antibody directed against activated caspase-3 (R&D Systems Europe) for 1 hour at 37°C, followed by an Alexa Fluor 488-conjugated goat anti-rabbit secondary antibody (Invitrogen). Cells treated with 0.5 µM staurosporine (Sigma-Aldrich) for 20 hours served as positive controls for apoptosis.

### Ammonia assay

Two µg/milliliter H*. suis* GGT was added to HBSS supplemented with 2 mM L-Gln and incubated at 37°C for 2 hours, after which the concentration of released ammonia was determined by the Ammonia Assay Kit (Sigma-Aldrich) according to the manufacturer’s instructions. 

### Supplementation of cell cultures with L-Gln and GSH

Jurkat T cells (4 × 10^4^/well) were incubated in medium supplemented with L-Gln (0 - 10 mM; Sigma-Aldrich) or GSH (0 - 5 mM; Sigma-Aldrich) and treated with HBSS or 2 µg/mL recombinant *H. suis* GGT for 48 or 72 hours. CD4^+^ and CD8^+^ T cells (1.2 × 10^5^/well) were incubated in medium supplemented with L-Gln (0 - 5 mM) or GSH (0 - 2 mM) and treated with HBSS or 1 µg/mL recombinant *H. suis* GGT for 68 hours. Cellular proliferation was determined by [^3^H]-thymidine incorporation as mentioned above.

### Measurement of cytokine release

CD4^+^ T cells (1.5 × 10^5^/well), activated by CD3/CD28 mAbs, were incubated in medium supplemented with 0.1 µg/mL or 0.5 µg/mL recombinant *H. suis* GGT for 68 hours. Secretion levels of IFN-γ, IL-4, and IL-17A were determined in cell supernatant by enzyme-linked immunosorbent assay (ELISA) (eBioscience). 

### Statistical analysis

All experiments were repeated at least 3 times with at least 3 replications for each treatment. Combined data from these experiments are used for statistical analysis, and all data were expressed as mean + SD. A Student *t* test was used for statistical analysis between two groups, and one-way ANOVA was performed for comparison of control cells with multiple treatments. For both statistical analyses methods, *P* values less than 0.05 were considered statistically significant.

## Results

### *H. suis* OMV contain GGT activity and can be internalized by AGS, IPEC-J2, and Caco-2 cells

**Figure 1:**
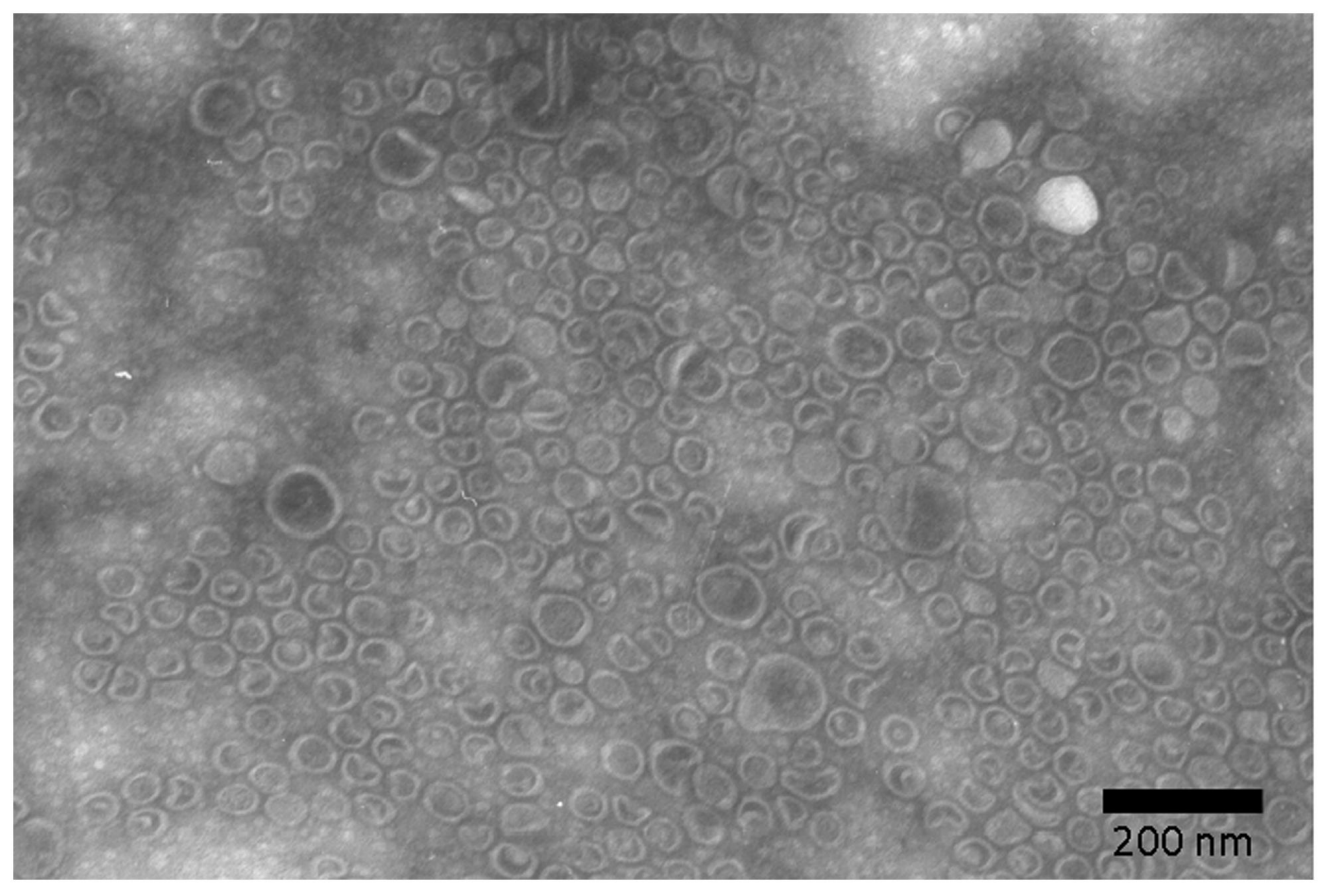
Ultrastructural examination of purified *H. suis* outer membrane vesicles (OMV). Shown are transmission electron microscopic images of *H. suis* OMV purified by repeated ultracentrifugation.

**Figure 2:**
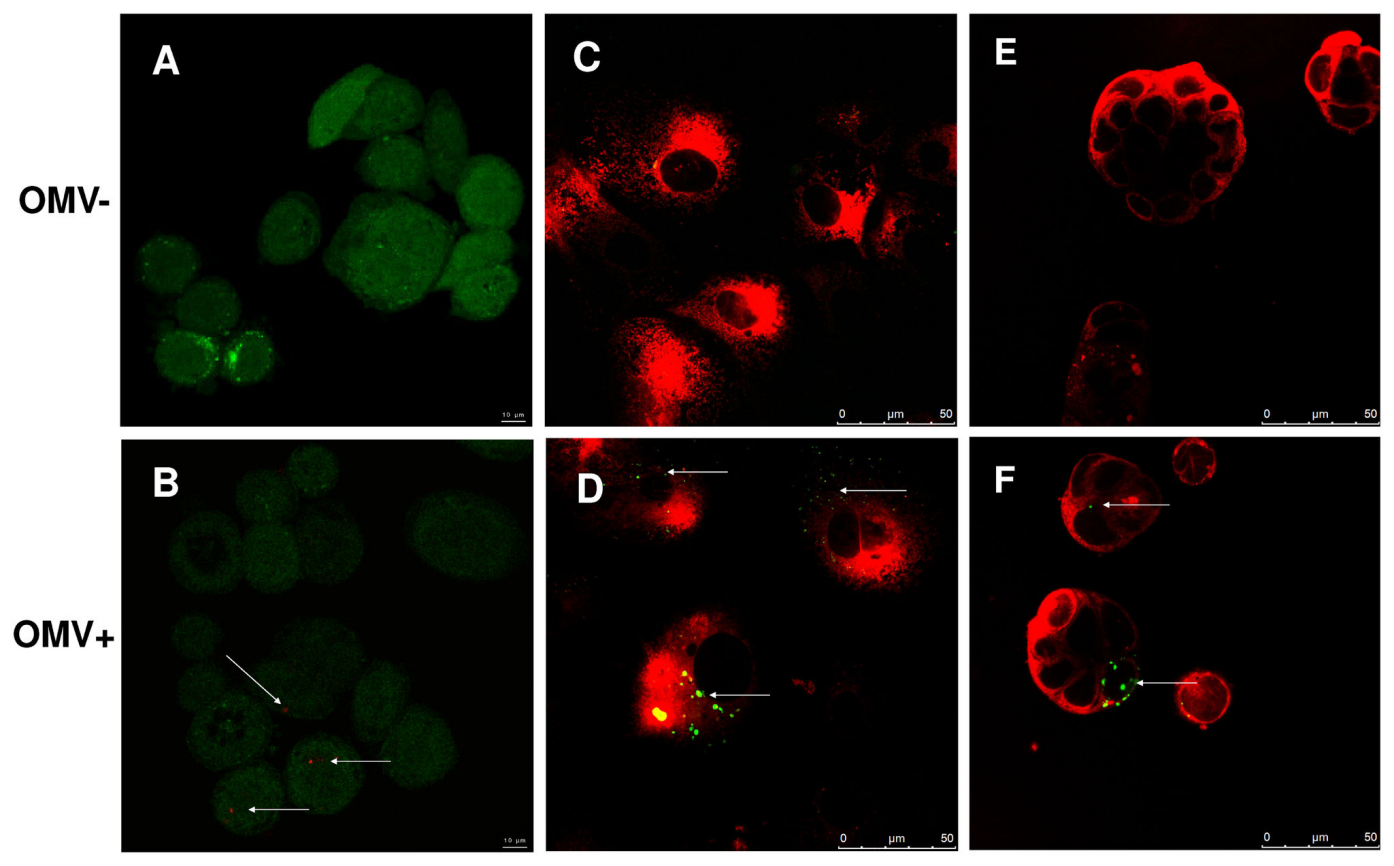
The uptake of *H. suis* OMV by AGS, IPEC-J2, and Caco-2 cells. AGS cells labeled with green CellTrackerTM were incubated for 4 hours with HBSS (Figure 2A) or *H. suis* OMV labeled with red fluorescent Vybrant® DiD (Figure 2B). IPEC-J2, and Caco-2 cells labeled with red fluorescent CellTracker Red CMTPX were incubated for 8 hours with HBSS (Figure 2C, 2E, respectively) or *H. suis* OMV labeled with green fluorescent Vybrant® DiO (Figure 2D, 2F, respectively). The visualization of OMV was done by confocal laser scanning microscopy (indicated by arrows). HBSS: Hank’s balanced salt solution; *H. suis* OMV: *Helicobacter. suis* outer membrane vesicles.

Ultrastructural examination revealed that most OMV isolated from *H. suis* culture supernatant ranged from 20 - 200 nm in size (Figure 1). A GGT activity level up to 4.5 - 9.5 mU/mg was detected in the OMV, confirming that GGT is one of the components of *H. suis* OMV. In order to further examine if *H. suis* OMV carrying GGT can be internalized by gastric or intestinal epithelial cells, AGS, IPEC-J2, and Caco-2 cells were incubated with *H. suis* OMV for 4 hours or 8 hours. Our results reveal that *H. suis* OMV can be internalized by all three types of epithelial cell lines (Figure 2 A - F). 

### Active *H. suis* GGT from *H. suis* OMV translocates across a differentiated IPEC-J2 cell monolayer

**Figure 3:**
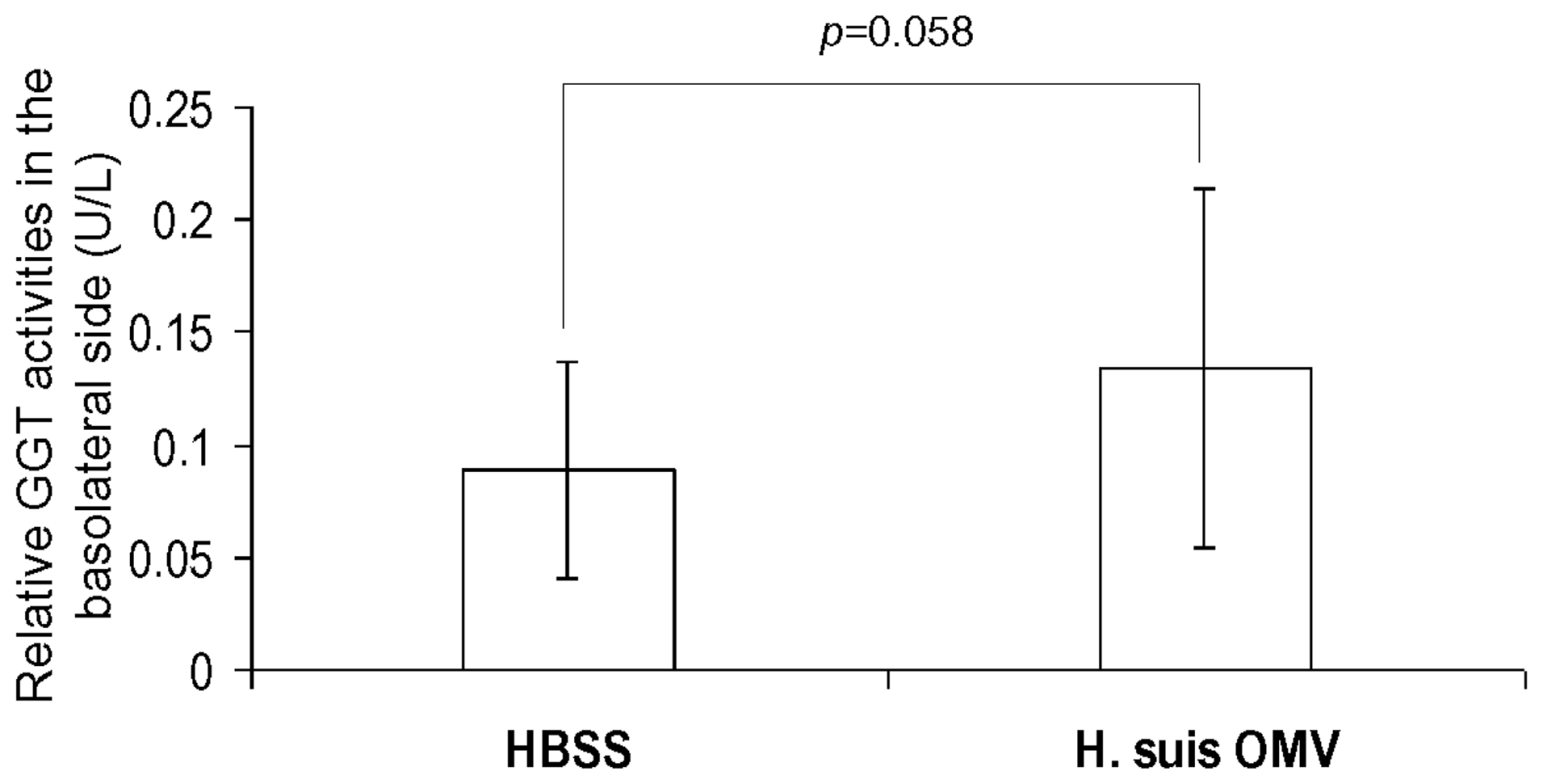
Translocation of active *H. suis* GGT through a differentiated IPEC-J2 cell monolayer. IPEC-J2 cells were seeded on the insert (with a pore size of 3.0 μm and a membrane diameter of 6.5 mm) for 3 - 4 weeks until the cells were differentiatd to a complete cell monolayer. The cells were treated with HBSS or 100 µg *H. suis* OMV for 48 hours, and the presence of GGT in the baselateral compartment was determined by a GGT activity assay as described before. Results are presented as the relative GGT activity level compared to control cells treated with HBSS. Shown are the mean values (± SD) of 3 independent experiments (n=9). Student *t* test was used for analysis of statistically significant difference. HBSS: Hank’s balanced salt solution; *H. suis* OMV: *Helicobacter. suis* outer membrane vesicles.

After 3 - 4 weeks culture, a differentiated IPEC-J2 cell monolayer was established, indicated by a stable TEER value of approximately 2400 Ohm/insert. Compared to the IPEC-J2 cells treated with HBSS, incubation of a differentiated IPEC-J2 cell monolayer with 100 µg *H. suis* OMV for 48 hours resulted in the detection of higher GGT activity in the basolateral compartment (Figure 3, *p*=0.058) without disrupting the integrity of IPEC-J2 cell monolayer, as shown by a stable TEER: an average value of 2421 Ohm/insert was detected at the onset of the experiment and an average value of 2361 Ohm/insert was detected at the end of the experiment (*p*=0.72, Student *t* test). This translocation may constitute one of the routes by which GGT from *H. suis* can access lymphocytes residing in the lamina propria underneath the lining epithelium. 

### Effect of *H. suis* whole-cell lysate on Jurkat T cells

**Figure 4:**
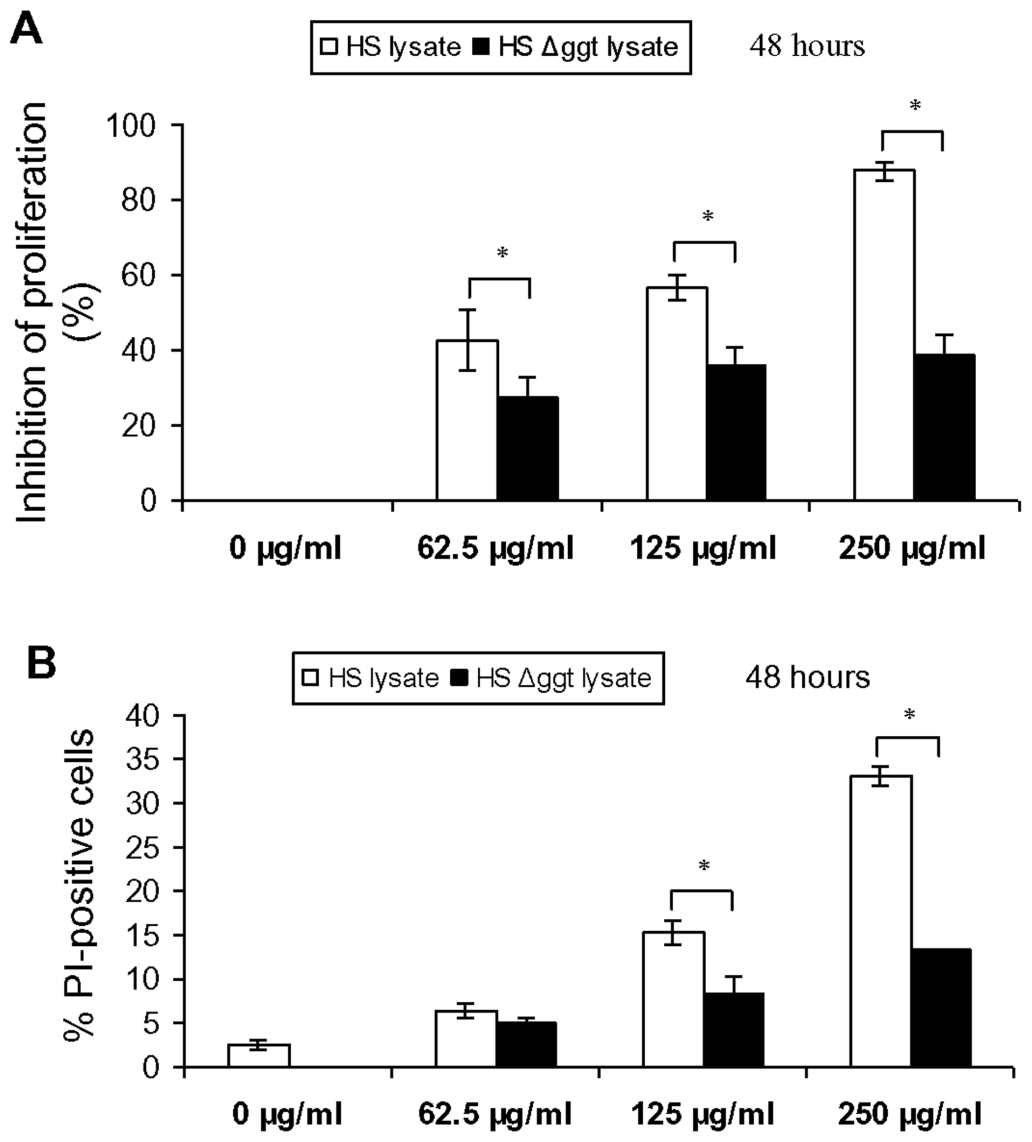
Effect of *H. suis* whole-cell lysate on cell proliferation and viability of Jurkat T cells. (*A*) Jurkat T cells were incubated in medium supplemented with whole-cell lysate (62.5 to 250 µg/mL) from wild-type *H. suis* strain HS5 and strain HS5Δ*ggt* for 48 hours, and cell proliferation levels are determined by cpm (counts per minute), as a measure of [^3^H]-thymidine uptake. Shown are the rates of proliferation inhibition, relative to Jurkat T cells treated with HBSS instead of whole-cell lysate. Both whole-cell lysate from *H. suis* strain HS5 and strain HS5Δ*ggt* induced a statistically significant inhibition of T cell proliferation, although this was far less pronounced for the mutant strain (one-way ANOVA). (*B*) Jurkat T cells were incubated in medium supplemented with whole-cell lysate (62.5 to 250 µg/mL) from *H. suis* strain HS5 and strain HS5Δ*ggt* for 48 hours, and loss of plasma membrane integrity (as a marker for necrosis) was determined by PI staining. Both whole-cell lysate from *H. suis* strain HS5 and strain HS5Δ*ggt* induced a statistically significant increase of PI-positive cells (one-way ANOVA), although this was far less pronounced for the mutant strain. Shown in *A* and *B* are the mean values (± SD) of 3 independent experiments (n=9). An * represents a statistically significant difference (*p* < 0.05) between HS lysate- and HS Δ*ggt* lysate-treated cells. Control: Jurkat T cell treated by Hank’s balanced salt solution. HS lysate: *H. suis* strain 5 lysate. HS Δ*ggt* lysate: *H. suis* strain HS5Δggt lysate.

Cellular proliferation of Jurkat T cells was inhibited after incubation with whole cell lysate of wild-type *H. suis* strain HS5 for 48 or 72 hours in a dose-dependent manner (data not shown). Concentrations of 250 µg/mL of this lysate almost completely inhibited cellular proliferation of Jurkat T cells (Figure 4A). Compared to treatment with whole-cell lysate from wild-type *H. suis* strain HS5, treatment of Jurkat T cells with lysate (48 h; 62.5 to 250 µg/mL) from strain HS5Δ*ggt* resulted in a marked decrease (minus 15.3 - 49.3%) of the inhibitory effect on T cell proliferation (Figure 4A). 

### Inhibitory effect of *H. suis* GGT on Jurkat T cells and mouse splenocyte subsets

**Figure 5:**
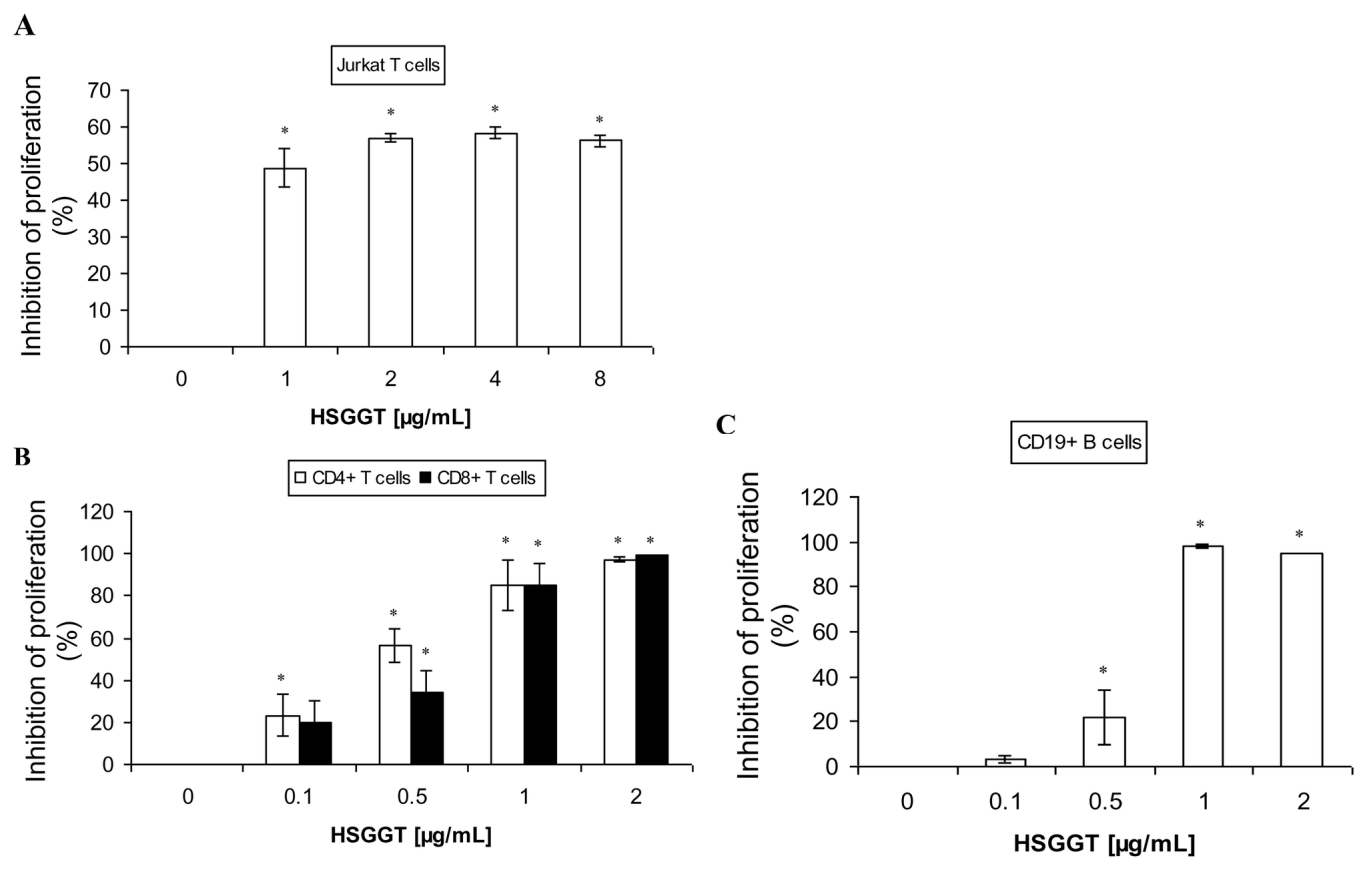
Inhibitory effect of *H. suis* γ-glutamyl transpeptidase (GGT) on Jurkat T cells and mouse splenocyte subsets. (*A*) Jurkat T cells were incubated in medium supplemented with recombinant *H. suis* GGT (1 to 8 µg/mL) for 72 hours, and cell proliferation levels are determined by cpm (counts per minute), as a measure of [^3^H]-thymidine uptake. (*B*) CD4^+^ or CD8^+^ splenic T lymphocytes were purified, stimulated by CD3/CD28 mAbs, and incubated with recombinant *H. suis* GGT (0.1 µg/mL to 2 µg/mL) for 68 hours, resulting in a dose-dependent inhibition of proliferation. (*C*) CD19^+^ B splenocytes were purified, stimulated by anti-IgM (12 µg/mL) and recombinant mouse IL-2 (100 U/mL), followed by treatment with recombinant *H. suis* GGT (0.1 µg/mL to 2 µg/mL) for 44 hours. Shown are the rates of proliferation inhibition, relative to stimulated splenocytes treated with HBSS instead of recombinant *H. suis* GGT. Shown are the mean values (± SD) of 3 independent experiments or one representative experiment (out of 3 performed in total). An * represents a statistically significant difference (*p* < 0.05) compared to HBSS-treated control cells. HSGGT: recombinant *H. suis* GGT.

Treatment of Jurkat T cells for 72 hours with up to 2 µg/mL recombinant *H. suis* GGT resulted in an inhibition of cellular proliferation (Figure 5A). Treatment for 48 hours showed similar results (data not shown). Further increasing the concentration of the enzyme, however, did not cause a significant increase of the inhibitory effect. Subsequently, we investigated the effect of recombinant *H. suis* GGT on primary immune cells, including CD4^+^ and CD8^+^ T cells and CD19^+^ B lymphocytes. A concentration of 1 µg/mL recombinant *H. suis* GGT inhibited the proliferation of CD4^+^ and CD8^+^ T splenocytes by about 80% (Figure 5B) and the proliferation of the CD19^+^ B cells by more than 95% (Figure 5C). A concentration of 2 µg/mL recombinant *H. suis* GGT almost completely inhibited the proliferation of all three lymphocyte subsets.

### The role of cell death (apoptosis and necrosis) during *H. suis* GGT-mediated inhibition of T cell proliferation

Compared to treatment with whole-cell lysate from wild-type *H. suis* strain HS5, treatment of Jurkat T cells with lysate (48 h; 62.5 to 250 µg/mL) from strain HS5Δ*ggt* resulted in a considerably lower (1.3 - 19.6%) cell death-inducing capacity (Figure 4B).

**Figure 6:**
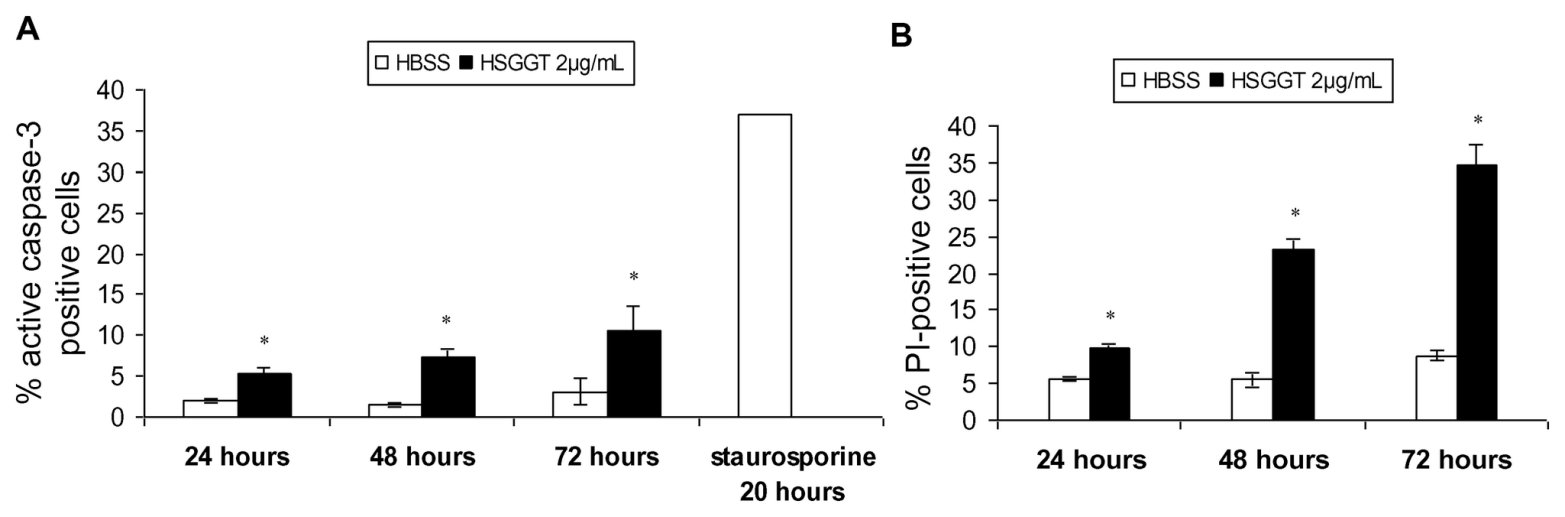
Cell death analysis of Jurkat T cells treated with recombinant *H. suis* γ-glutamyl transpeptidase (GGT) evaluated by flow cytometry. Jurkat T cells were incubated in medium supplemented with 2 µg/mL recombinant *H. suis* GGT for 24, 48, or 72 hours and (*A*) cell apoptosis and (*B*) loss of plasma membrane integrity (as a marker for necrosis) were determined by staining for activated caspase-3 and PI staining, respectively. Jurkat T cells treated with 0.5 µM staurosporine for 20 hours served as positive control for apoptosis. Shown are the mean values (± SD) of one representative experiment (n=3) or 3 independent experiments (n=9). An * represents a statistically significant difference (*p* < 0.05) between HSGGT- and HBSS-treated cells (Student *t* test). HBSS: Hank’s balanced salt solution; HSGGT: recombinant *H. suis* GGT; PI: propidium iodide.

Compared to the HBSS-treated cells, incubating Jurkat T cells with 2 µg/mL recombinant *H. suis* GGT for 24, 48 or 72 hours resulted in an increase (+3 - 7%) of the number of active caspase-3 positive cells (Figure 6A). PI staining demonstrated a higher increase (+26%, compared to HBSS-treated cells) of the number of Jurkat T cells showing loss of plasma membrane integrity, as a marker for necrosis, after treatment with *H. suis* GGT for 72 hours (Figure 6B). 

### Identification of catalytic activity of *H. suis* GGT on L-Gln

**Figure 7:**
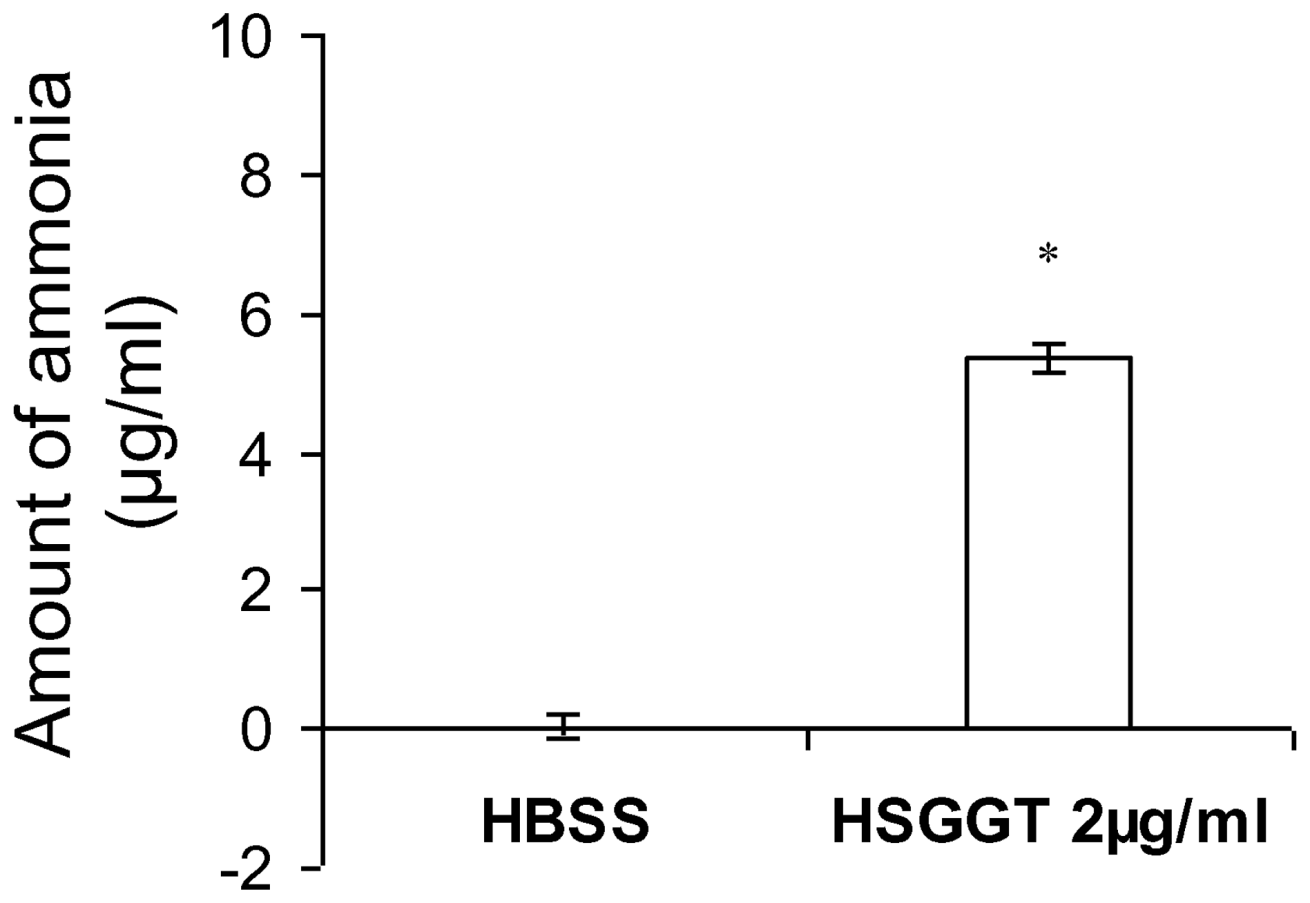
Determination of catalytic activity of *H. suis* GGT on **L-Gln**. Two mM Gln was incubated with HBSS or 2 µg/mL H*. suis* GGT at 37°C for 2 hours, after which the concentration of released ammonia was determined using the Ammonia Assay Kit. The mean data (± SD) of one representative experiment are shown (n=3). An * represents a statistically significant difference (*p* < 0.05) compared to HBSS-treated L-Gln (Student *t* test). HSGGT: recombinant *H. suis* GGT; HBSS: Hank’s balanced salt solution.

L-Gln and reduced glutathione (GSH) are 2 putative substrates of *H. suis* GGT. In a previous report, we indeed showed that *H. suis* GGT catalyzes the degradation of GSH [9]. To investigate whether also L-Gln can serve as a substrate for *H. suis* GGT, 2 mM Gln was incubated in HBSS with or without 2 µg/mL H*. suis* GGT at 37°C. After 2 hours of incubation the concentration of ammonia was determined. Data showed that *H. suis* GGT indeed hydrolyses Gln *in vitro*, with the formation of ammonia as by-product (Figure 7). Compared to HBSS-treated Gln, 2 mM Gln treated with 2 µg/mL H*. suis* GGT released 5.3 µg/mL ammonia after incubation for 2 hours, showing that >15% of Gln was degraded by 2 µg/mL H*. suis* GGT under these conditions. More than 70% of Gln was degraded by using a higher concentration of *H. suis* GGT (10 µg/mL), after incubation under the same conditions as described above (data not shown).

### Modulation of *H. suis* GGT-mediated inhibition of lymphocyte proliferation by L-Gln and GSH

**Figure 8:**
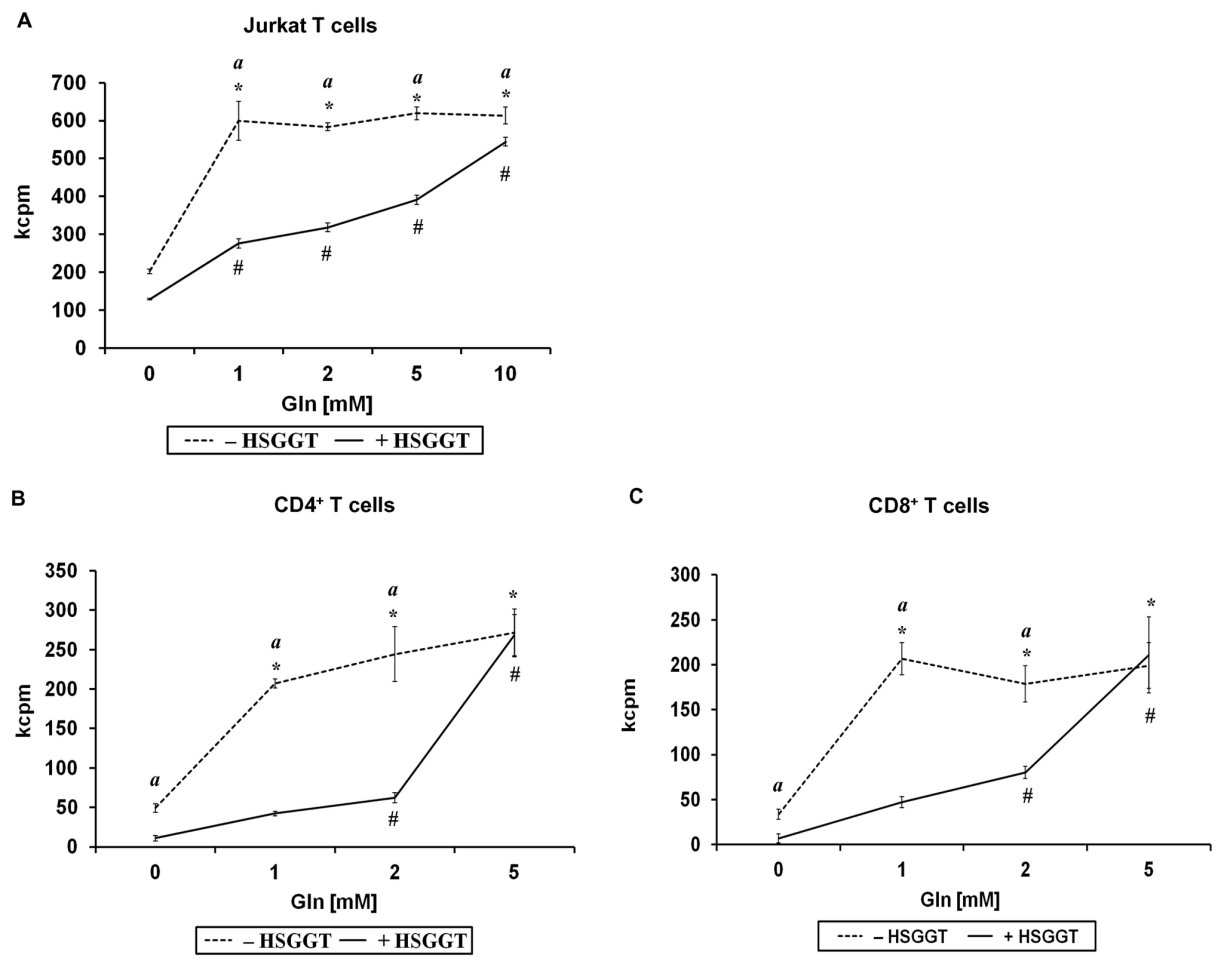
The role of L-Gln supplementation to *H. suis* γ-glutamyl transpeptidase (GGT)-treated Jurkat T cells and murine splenocytes. Jurkat T cells were incubated in medium supplemented with L-Gln (0 mM to 10 mM) for 72 hours (*A*) in the presence or absence of 2 µg/mL recombinant *H. suis* GGT, followed by cell proliferation detection by determining [^3^H]-thymidine uptake. CD4^+^ T cells (*B*) or CD8^+^ T cells (*C*) activated by anti-CD3 and anti-CD28 mAbs, were supplemented with L-Gln (0 mM to 10 mM) for 68 hours in the presence or absence of 1 µg/mL recombinant *H. suis* GGT, followed by cell proliferation detection by measuring [^3^H]-thymidine uptake. The mean data (± SD) of one representative experiment (out of 3 performed in total) are shown for *A-C* (n=3). * and # represent a statistically significant increase (*p* < 0.05 ) of cell proliferation by supplementing cells with a given L-Gln concentration, compared to HBSS- or *H. suis* GGT-treated cells, respectively, without L-Gln supplementation (0 mM L-Gln) (one-way ANOVA). An (*a*) indicates a higher proliferation rate of HBSS-treated cells, compared to *H. suis* GGT-treated cells for a given L-Gln concentration (Student *t* test). *, #, and (*a*): *p* < 0.05. kcpm: the number of counts per minute (x1000) determined by β-scintillation counting, as a measure of cellular proliferation; HSGGT: recombinant *H. suis* GGT; CD3/CD28 mAbs: anti-mouse CD3/CD28 monoclonal antibodies; Gln: L-glutamine; -HSGGT: treated without recombinant *H. suis* GGT; +HSGGT: treated with recombinant *H. suis* GGT.

To investigate the role of L-Gln and GSH, two important substrates of GGT, in the above described inhibition of lymphocyte proliferation, Jurkat T cells and stimulated CD4^+^ or CD8^+^ T cells isolated from mice, were treated with a series of concentrations of L-Gln or GSH in the presence or absence of 1 or 2 µg/mL recombinant *H. suis* GGT. Data from HBSS-treated control cells showed that the presence of L-Gln is essential for a normal proliferation of Jurkat T cells (Figure 8A). As described above, treatment of Jurkat T cells with recombinant *H. suis* GGT resulted in an inhibition of cellular proliferation. Interestingly, supplementation of 2 µg/mL H*. suis* GGT-treated Jurkat T cells with L-Gln was able to restore the normal proliferation rate of the cells, incubated for 72 hours (Figure 8A), in a dose (up to 10 mM L-Gln)-dependent manner. For primary CD4^+^ or CD8^+^ T lymphocytes isolated from mouse spleens, supplementation with L-Gln showed a similar effect (Figure 8B, 8C). Supplementation with 5 mM L-Gln was able to restore the cellular proliferation of 1 µg/mL recombinant *H. suis* GGT treated CD4^+^ and CD8^+^ T cells to normal levels after incubation for 68 hours (Figures 8B, 8C).

**Figure 9:**
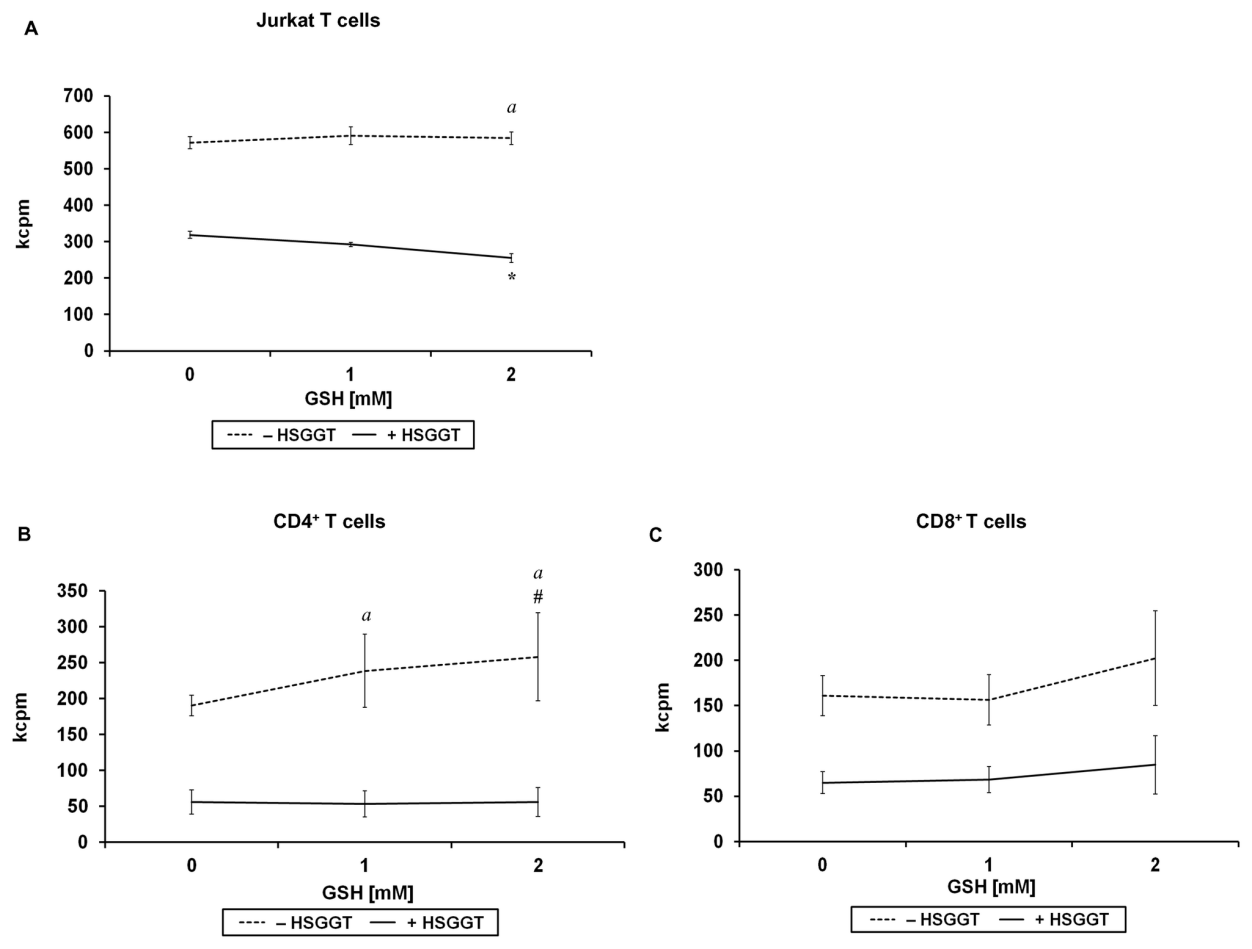
The role of GSH supplementation to *H. suis* γ-glutamyl transpeptidase (GGT)-treated Jurkat T cells and murine splenocytes. Jurkat T cells were incubated in medium supplemented with GSH (0 mM to 2 mM) for 72 hours (*A*) in the presence or absence of 2 µg/mL recombinant *H. suis* GGT, followed by cell proliferation detection by measuring [^3^H]-thymidine uptake. The mean data (± SD) of one representative experiment are shown (n=3). CD4^+^ T cells (*B*) or CD8^+^ T cells (*C*) activated by CD3/CD28 mAbs, were incubated in medium supplemented with GSH (0 mM to 2 mM) for 68 hours in the presence or absence of 1µg/mL recombinant *H. suis* GGT, followed by cell proliferation detection by measuring [^3^H]-thymidine uptake, as shown by kcpm (counts per minute; x1000) values. Shown are the mean values (± SD) of 3 independent experiments (n=9). An * indicates a decrease of cell proliferation of *H. suis* GGT-treated cells supplemented by a given GSH concentration, compared to *H. suis* GGT treated cells without GSH supplementation (0 mM GSH) (one-way ANOVA). An # indicates an increase of cell proliferation of HBSS-treated cells supplemented by a given GSH concentration, compared to HBSS-treated cells without GSH supplementation (0 mM GSH) (one-way ANOVA). An (*a*) indicates the relative increase of the difference of cell proliferation between HBSS-treated cells and *H. suis* GGT-treated cells at an indicated concentration of GSH, compared to 0 mM GSH-treated cells (Student *t* test). *, #, and (*a*): *p* < 0.05. kcpm: the number of counts per minute (x1000) determined by β-scintillation counting, as a measure of cellular proliferation; HSGGT: recombinant *H. suis* GGT; CD3/CD28 mAbs: anti-mouse CD3/CD28 monoclonal antibodies; GSH: reduced glutathione; -HSGGT: treated without recombinant *H. suis* GGT; +HSGGT: treated with recombinant *H. suis* GGT.

On the other hand, GSH supplementation induced a slightly higher stimulation of cellular proliferation of primary T splenocyte subsets (Figure 9B, P< 0.05), treated with HBSS (control cells). Interestingly and in contrast, supplementation of *H. suis* GGT-treated Jurkat T cells with GSH aggravated the inhibitory effect of *H. suis* GGT, both after 48 and 72 hours of incubation (Figure 9A, P< 0.05). For CD4^+^ or CD8^+^ T cells, however, we did not observe similar effects (Figure 9B, 9C).

### Effects of *H. suis* GGT on T helper cytokine secretion by murine CD4^+^ T cells

**Figure 10:**
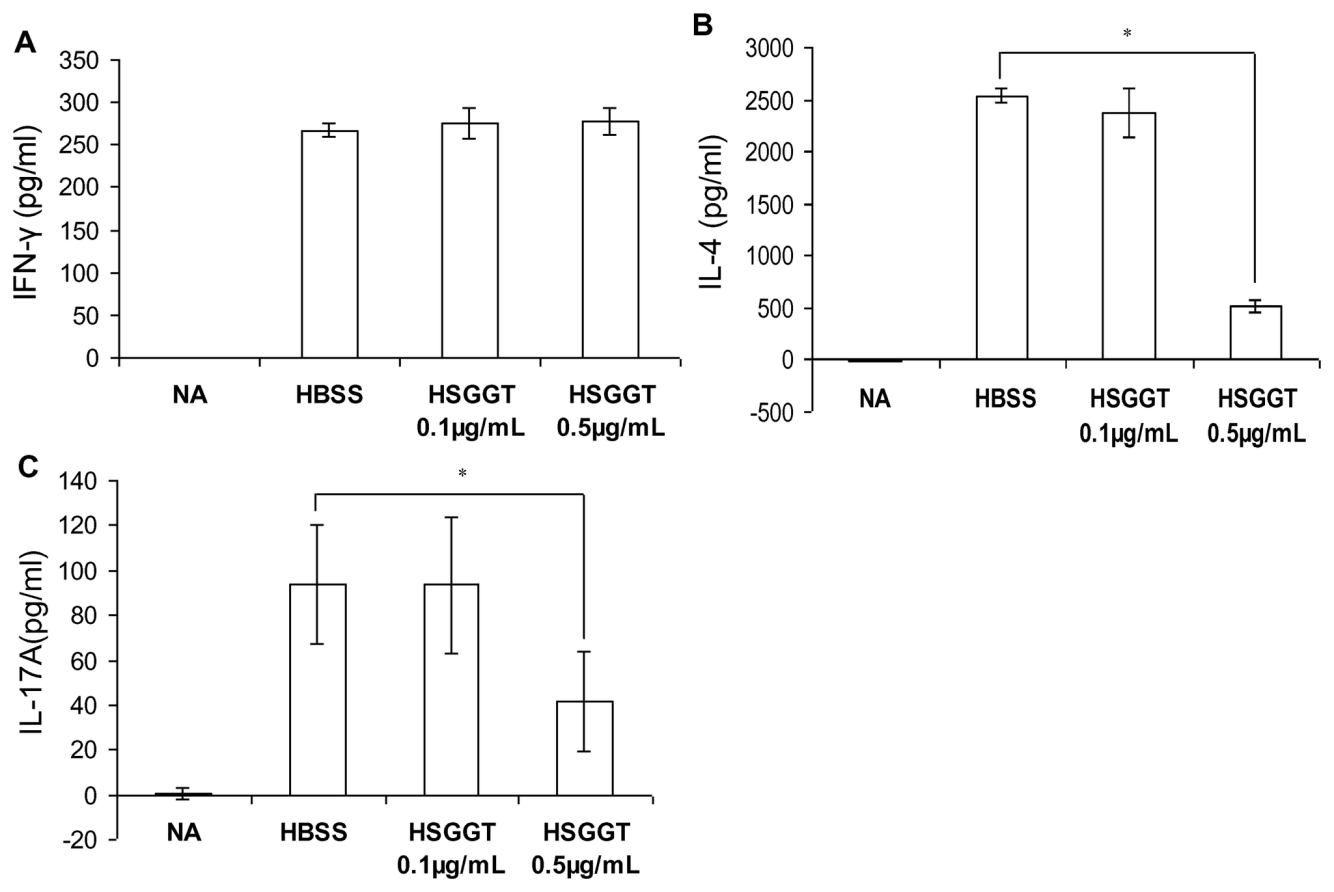
Influence of *H. suis* γ-glutamyl transpeptidase (GGT) on Th1, Th2, and Th17 type cytokine secretion by murine CD4^+^ T cells. IFN-γ (*A*), IL-4 (*B*), and IL-17A (*C*) secretion by CD4^+^ T cells activated by anti-CD3 and anti-CD28 mAbs was measured after 68 hours by enzyme-linked immunosorbent assay. Data represent mean ± SD of one representative experiment (n=4). * *p* < 0.05 (one-way ANOVA). NA: unstimulated CD4^+^ T cells in the absence of *H. suis* GGT; HBSS: stimulated CD4^+^ T cells in the absence of *H. suis* GGT; HSGGT: stimulated CD4^+^ T cells in the presence of recombinant *H. suis* GGT.

CD4^+^ T cells are known to play a pivotal role in the immune response directed against *Helicobacter* infection [41-43]. The results described above show that *H. suis* GGT inhibits the proliferation of this lymphocyte subset. We investigated whether this also implies a change in cytokine secretion by these cells. Murine CD4^+^ T cells were incubated with 0.1 µg/mL or 0.5 µg/mL recombinant *H. suis* GGT for 68 hours. Enzyme-linked immunosorbent assay (ELISA) for IFN-γ, IL-4 and IL-17A performed on supernatant fluids of these cells revealed a significant suppression of IL-4 and IL-17A secretion, a Th2 and Th17 signature cytokine, respectively, in the presence of 0.5 µg/mL recombinant *H. suis* GGT (Figure 10B, 10C, *P*< 0.05). For IFN-γ secretion by these same cell populations, however, no effects were observed upon treatment with *H. suis* GGT (Figure 10A). 

## Discussion

To date, limited information is available on the virulence mechanisms of *H. suis* [4]. The development, in 2008, of a method for *in vitro* isolation and culture of *H. suis*, facilitated research on the interactions between *H. suis* and its hosts [44]. In a previous study, *H. suis* was shown to cause a chronic infection, leading to severe gastric lesions in mouse and Mongolian gerbil models of human gastric disease [3]. For *H. pylori*, inhibition of lymphocyte proliferation is considered to contribute to the immune evasion of *H. pylori*, enabling the bacterium to establish a chronic infection [45,46]. Several *H. pylori* factors have been described to be involved in inhibition of T lymphocyte proliferation, including the *H. pylori* GGT [13,15,45,46]. Similarly, *H. bilis* GGT was reported to inhibit T cell proliferation at a similar level compared to *H. pylori*, and both *H. bilis* and *H. pylori* GGT possess a similar suppressive effect on gastric epithelial cell proliferation mediated by an apoptosis-independent mechanism [18]. In a recent study, we identified part of the mechanism by which *H. pylori* and *H. suis* GGT cause gastric epithelial cell death [17]. An important role was attributed to the extracellular cell-independent formation of prooxidant metabolites through *H. suis* GGT-mediated degradation of GSH [17]. In the present study, we investigated a potential effect of *H. suis* GGT on the proliferation of lymphocytes and more importantly demonstrated a possible role for degradation of its known substrates in this process. 

In the present study, recombinantly expressed *H. suis* GGT, as well as whole-cell lysate of wild type *H. suis* strain HS5 had an inhibitory effect on the proliferation of Jurkat T cells, whereas this effect was much lower when Jurkat T cells were incubated with whole-cell lysate of the isogenic *H. suis ggt* mutant strain HS5Δ*ggt*. Recombinantly expressed *H. suis* GGT also inhibited the proliferation of different subsets of primary mouse lymphocytes and these effects were more pronounced than those observed in Jurkat T cells, since in primary splenocytes, 0.1 µg/mL H*. suis* GGT already caused a detectable inhibitory effect. However, using different concentrations of whole-cell lysate from strain HS5Δ*ggt* did not completely abolish the inhibitory effect on Jurkat T cell proliferation, suggesting that other factors are also involved. Putative virulence factors of *H. suis* other than GGT contributing to the inhibition of lymphocyte proliferation need to be further investigated in future experiments.

In Jurkat T cells, *H. suis* GGT-mediated inhibition of proliferation was correlated with an increase of both apoptosis and necrosis. Apparently, this is in contrast to what has been described for *H. pylori* GGT, which does not seem to induce apoptosis in Jurkat T cells, although it has to be mentioned that no other types of cell death were investigated in the study by Schmees et al. [15]. On the other hand, in a previous study, we demonstrated that *H. suis* GGT can induce death of gastric epithelial cells, both by necrosis/oncosis and apoptosis, depending on the amount of extracellular reactive oxygen species, generated by GSH degradation[17]. Most likely, these increased concentrations of reactive oxygen species in the extracellular environment are also involved in causing death of Jurkat T cells. 

In the supernatant of a 24-hour-old to 48-hour-old *H. suis* culture (containing 1 - 4 x 10^8^ bacteria/mL with a viability of >99%), approximately 2 - 5 mU/mL GGT activity can be detected [17]. Currently, no exact data are available on the colonization density of *H. suis* in human stomachs. Average numbers of *H. suis* colonizing the stomach of experimentally infected mice can reach approximately 10^8^ - 10^9^/g tissue [37] and 10^8^/g tissue in the stomach of experimentally as well as naturally infected pigs, with colonization densities as high as 10^10^ - 10^11^/g tissue in some cases [unpublished results]. These values thus correspond in general to the numbers of bacteria per mL in *in vitro* cultures, as mentioned above. Extrapolation clearly shows that the amounts of *H. suis* lysate or GGT used in the current study most likely are similar to what can be expected to be present in vivo. Indeed, *H. suis* lysate (containing 25 mU GGT activity/mg total protein) [17] was added to the Jurkat T cells at a final concentration of 62.5 to 250 µg/mL to reach a final concentration of 1.5 to 6.25 mU/mL GGT activity. Recombinant *H. suis* GGT (containing 8 mU GGT activity/µg purified *H. suis* GGT) was added to Jurkat T cells and murine splenocyte subset cultures at a final concentration of 0.1 to 2 µg/mL to reach similar levels of GGT activity (0.8 to 16 mU/mL) in the supernatant fluid of the cells.

As shown in the present and previous studies, L-Gln and GSH are two important substrates of GGT enzymes, including that of *H. suis* [17,18,47]. The present report is the first one describing that the effects induced by *H. suis* GGT on the function of lymphocytes can be largely attributed to its catalytic activity on extracellular L-Gln and GSH. As GGT activity and function are considered to be conserved among the genus *Helicobacter* [18], similar effects can be expected for GGT from other helicobacters. 

L-Gln is the most abundant free amino acid in the blood, and is in fact a major fuel for immune cells, especially lymphocytes [48-50]. Sufficient L-Gln is essential for both a complete proliferation capacity and normal immune functions of T lymphocytes [51,52]. In addition, several reports indicate that L-Gln supplementation has a general protective effect on eukaryotic cells, especially lymphocytes [53-55]. Treatment of lymphocytes with *H. suis* GGT, as in the present study, causes a depletion of extracellular L-Gln, due to the deamidation of L-Gln to L-glutamate (L-Glu), with formation of ammonia as a by-product [19,56]. Results of the present *in vitro* study also show that supplementation of *H. suis* GGT-treated lymphocyte cultures with a series of concentrations of L-Gln strongly counterbalances the inhibitory effect of *H. suis* GGT, stressing the importance of this amino acid for the proliferation of lymphocytes. 

It has been extensively studied and accepted that the mammalian intestine can absorb and utilize L-Gln both from the bloodstream as well as the intestinal lumen [21,57-60]. Little information is available on the L-Gln transport or utilization by epithelial cells or other cell types in the gastric mucosa [61,62]. Transcripts from several amino acid transporter systems for L-Gln have been shown to be expressed in murine and human stomach tissue, including amino acid transporter systems N, A, and L [60,63-66]. In any case, when Gln is partially delivered to lymphocytes from the gastrointestinal lumen, the link between Gln depletion and the GGT from *H. suis* (as well as other gastric *Helicobacter* species), is obvious, since the GGT can easily access the free Gln in the lumen. On the other hand, it is believed by many researchers that the GGT from gastric helicobacters as well as other secreted factors such as the VacA from *H. pylori* can access the lymphocytes in the lamina propria, in this way affecting the lymphocyte function in a direct and indirect manner. This can be achieved by inflicting damage to epithelial cells, causing local defects in the epithelial barrier [3,17,25,26,67]. In the present study, we have also provided data supporting our hypothesis that the active GGT enzyme from *H. suis* can cross a differentiated epithelial cell layer, in this way reaching the Gln (and GSH) provided to lymphocytes residing in the lamina propria. We were able to show that the active GGT is one of the components of OMV of *H. suis*, and that the OMV can be internalized, resulting in a translocation of the active *H. suis* GGT from the apical to the basolateral side of epithelial cells, enabling the GGT to locally access the nutrients (eg. Gln) provided from the arterial blood flow. 

*In vivo*, gastric helicobacters induce a deamidation of extracellular L-Gln to L-Glu, after which the latter can be taken up by the bacteria [56], depriving host epithelial and immune cells from both amino acids [26]. In the present study, no viable bacteria were used, capable of using extracellular L-Glu. Therefore, no depletion of L-Glu is instilled under the experimental conditions described in this study. In theory, L-Glu could thus serve as an alternative cellular fuel, replacing L-Gln, since both amino acids have been described to be able to serve as a cellular fuel for lymphocytes and gastrointestinal epithelial cells [51,68-70]. The fact that L-Glu can not simply replace L-Gln with respect to cellular proliferation, most likely depends on the wider array of functions of L-Gln. For instance, L-Gln, but not L-Glu, can be used for purine and pyrimidine synthesis [68], and L-Gln is involved in regulation of protein turnover [57]. Possibly, some relevant pathways mentioned above are also involved in T cell proliferation modulated by *H. suis* GGT and L-Gln.

GSH, another substrate for *H. suis* GGT, is considered to be the most important free thiol in animal cells, playing an important role in antioxidant defense, nutrient metabolism, and regulation of cellular events [71,72]. However, several groups have also described pro-oxidative reactions associated with the metabolism of extracellular GSH, initiated by GGT, which may lead to the production of reactive oxygen species and lipid peroxidation, followed by cell death or inhibition of cellular proliferation [17,20,72,73]. Large amounts of intracellular and extracellular GSH, indeed available in the stomach [17], may act as a substrate to GGT during *H. suis* infection. In the present study, we showed that supplementation with GSH could enhance the proliferation of untreated control Jurkat T cells and murine T lymphocytes to a certain extent. In sharp contrast, when supplementing GSH to *H. suis* GGT-treated Jurkat lymphocytes, this even aggravated *H. suis* GGT-induced inhibition of cell proliferation, possibly due to the pro-oxidative effect of GSH metabolites. However, supplementing GSH to *H. suis* GGT-treated primary mouse lymphocytes, caused no aggravation of *H. suis* GGT-induced inhibition of cell proliferation. Possibly, primary mouse lymphocytes are less sensitive to pro-oxidative products formed under the current experimental conditions, compared to the human-derived Jurkat cell line. Most likely, the balance between concentrations of the antioxidant GSH and its pro-oxidative degradation products is important. Further investigation in primary mouse lymphocytes, using different concentrations of *H. suis* GGT and/or GSH, will allow us to determine whether an effect similar to that seen in Jurkat cells occurs.

IFN-γ, IL-4 and IL-17A are considered to be signature cytokines secreted by T helper (Th) 1, Th2 or Th17 cells, respectively [74]. In the present study, IFN-γ secretion by activated CD4^+^ T cells seems unaffected by *H. suis* GGT treatment, whereas *H. suis* GGT treatment did inhibit IL-4 and IL-17A secretion by activated CD4^+^ T cells, showing that the effects of *H. suis* GGT on the proliferation of CD4^+^ helper T lymphocytes also affect the functional secretion of cytokines involved in the maintenance of an immune response. 

 In summary, *H. suis* GGT was found to inhibit the proliferation of lymphocytes, making it the first discovery of a virulence factor of *H. suis* that affects the functions of immune cells. Cell death plays an important role in this process. Supplementation of *H. suis* GGT-treated lymphocytes with L-Gln or GSH was able to modulate the observed inhibitory effect, however in opposite ways. L-Gln was able to restore the normal proliferation of the cells whereas supplementation with reduced glutathione (GSH) aggravated the inhibition of lymphocyte proliferation induced by *H. suis* GGT. In addition, we demonstrated that the inhibition of T cell proliferation by *H. suis* GGT is not identical for different lymphocyte subsets, and that *H. suis* GGT also affects the cytokine secretion of CD4^+^ lymphocytes. Finally, we have generated data supporting our hypothesis that the uptake and processing of *H. suis* OMV by epithelial cells may result in the delivery of active *H. suis* GGT to lymphocytes residing in the deeper mucosal layers. The above described findings may explain part of the mechanisms by which *H. suis* establishes a chronic infection in its preferred niche.
