# Correction: Effects of *Helicobacter suis* γ- Glutamyl Transpeptidase on Lymphocytes: Modulation by Glutamine and Glutathione Supplementation and Outer Membrane Vesicles as a Putative Delivery Route of the Enzyme

**DOI:** 10.1371/annotation/3af92252-efe3-426b-bf1b-b90d29f57b53

**Published:** 2014-01-06

**Authors:** Guangzhi Zhang, Richard Ducatelle, Frank Pasmans, Katharina D’Herde, Liping Huang, Annemieke Smet, Freddy Haesebrouck, Bram Flahou

The last two authors, Freddy Haesebrouck and Bram Flahou, should be noted as sharing senior authorship. 

